# Prognostic scores and infective endocarditis

**DOI:** 10.1186/cc11980

**Published:** 2013-03-19

**Authors:** P Fernandez Ugidos, R Gomez Lopez, P Vidal Cortes, A Ceniceros Barros, L Seoane Quiroga, JM Lopez Perez

**Affiliations:** 1 Complejo Hospitalario Universitario Ourense, Spain; 2 Complejo Hospitalario Universitario A Coruña, Spain

## Introduction

Infective endocarditis (IE) is a high-mortality disease, especially in the early surgery subgroup. The aim of this study was try to identify prognostic factors of IE that will require surgery during the same admission of their diagnosis, including evaluation of surgical severity scores.

## Methods

A retrospective study (5 years) of all patients admitted to a tertiary hospital in northern Spain with diagnosis of IE (modified Duke criteria) who required early surgery. Demographic, clinical and microbiology data were collected. Chi-square and Student *t *tests. Significance: *P *< 0.05. SPSS17.

## Results

We had 73 patients, 79.5% men, age 65 years. Eighty-two percent had positive blood cultures. Forty-one percent of cases required previous ICU admission. Surgery was urgent in 35%. Fifty-six per cent of patients had postoperative shock and 58% suffered post-surgery ARF. Hospital mortality was 31.5%. Regarding prognostic scales: the mean EuroSCORE was 11.38 ± 3.93 points. No patient was placed in the low-risk group. Ninety-four per cent of cases were at high risk. The Parsonnet mean score was 27.7 ± 11.6. The mean Beth Israel Medical Center was 37.6 ± 10 points. The mean Ontario scale was 7.09 ± 2.7. The mean Surgical risk scale of Roques was 6.69. And the mean by Pons Scale was 24.02. Dead patients are older, with previous heart disease, require urgent surgery and have previous ICU stay; usually they have MOF and they had received less than 7 days of antibiotic treatment. The etiology did not worsen the prognosis.

## Conclusion

In early surgery IE, it appears to be associated with mortality: age >70 years, previous heart disease, emergency surgery, antibiotics within 7 days before surgery, preoperative MOF, and high scores on scales of Pons and Ontario. The causal agent and echocardiography have no relation with worse prognosis. The general syndrome debut and the Streptococcus spp. etiology seem to have lower mortality. IE has pathologies with high preoperative severity scores but these are not sufficient to guide therapeutic decisions.

